# Genetic polymorphisms in ataxin-3 and liver cirrhosis risk related to aflatoxin B1

**DOI:** 10.18632/oncotarget.24535

**Published:** 2018-02-19

**Authors:** Xing-Zhizi Wang, Xiao-Ying Huang, Jin-Guang Yao, Chao Wang, Qiang Xia, Xi-Dai Long

**Affiliations:** ^1^ Department of Pathology, The Affiliated Hospital of Youjiang Medical University for Nationalities, Baise 533000, China; ^2^ Department of Digestive Medicine, The Affiliated Hospital of Youjiang Medical University for Nationalities, Baise 533000, China; ^3^ Department of Liver Surgery, Ren Ji Hospital, School of Medicine, Shanghai Jiao Tong University, Shanghai 200127, China; ^4^ Guangxi Clinic Research Center of Hepatobiliary Diseases, Baise 533000, China

**Keywords:** ataxin-3, polymorphism, liver cirrhosis, aflatoxin B1, Gerotarget

## Abstract

**Background:**

Altered expression of ataxin-3 (AT3) can modify DNA repair capacity and is observed in human diseases. The genetic polymorphisms of this gene in aflatoxin B1 (AFB1)–related liver cirrhosis (LC) have not yet been elucidated.

**Materials and Methods:**

We conducted a hospital-based case–control study, including 384 patients with LC and 851 controls without any liver diseases, to assess the association between 264 polymorphisms in AT3 and AFB1-related LC risk. Genotype were tested using TaqMan-PCR or sequencing technique.

**Results:**

We found three differentially distributed SNPs (rs8021276, rs7158733, and rs10146249) via the screening analysis; however, only rs8021276 polymorphism was further identified to modify the risk of LC. Compared with the homozygote of rs8021276 A alleles (rs8021276-AA), the genotypes of rs8021276 G alleles (rs8021276-AG or -GG) increased LC risk (OR: 2.48 and 6.98; 95% CI: 1.84–3.33 and 4.35–11.22, respectively). Significant interactive effects between risk genotypes and AFB1 exposure status were also observed in the joint effects analysis. Additionally, rs8021276 polymorphism was also associated with down-regulation of AT3 mRNA expression and increasing AFB1-DNA adducts in liver tissues with cirrhosis.

**Conclusions:**

These results suggest AT3 polymorphisms may be risk biomarkers of AFB1-related LC, and rs8021276 is a potential candidate.

## INTRODUCTION

Aflatoxin B1 (AFB1) exposure is prevalent in the Bose population and is associated with a variety of clinical consequences [1, 2]. Some persons with chronic AFB1 exposure are in the diseases-free state; whereas others develop chronic liver damage including liver cirrhosis, which finally develops into hepatocellular carcinoma (HCC) [1–3]. Increasing evidence have shown that persistent exposure with AFB1 can induce liver injury, fibrosis connective tissue proliferation, and ultimately result to liver cirrhosis [4–6]. However, only a minority of lifelong chronic exposed individuals will eventually develop liver cirrhosis, and the cellular and molecular mechanisms of liver cirrhosis pathogenesis are still not completely understood. In the context of chronic liver damage, genetic polymorphisms, such as the single-nucleotide polymorphisms (SNPs), have been considered a risk factor for liver cirrhosis [7, 8].

Ataxin-3 (AT3) is an important deubiquitylation enzyme (DUB) encoded by the corresponding gene located on chromosome 14q21 [9]. Increasing evidence have shown that AT3 expression can express more than fifty-six alternatively spliced messenger RNAs (mRNAs) and 20 of these spliced mRNAs can translate into corresponding functional proteins. Its full-long expressing product possesses 376 amino acids and 42 kilodaltons of molecular weight [9, 10]. AT3 protein consists of two functional regions: (1) a globular Josephin domain with deubiquitinating activity in the N-terminal (the 1st to 170th amino acid) and (2) a flexible functional tail in the C-terminal (FFTC) that contains two ubiquitin-interacting motifs (located at the 223th to 240th and the 243th to 260th amino acid, respectively), a casein kinase II site (the 227th to 280th amino acid), nuclear localization signal site (the 282th to 285th amino acid), and a polyglutamine structure (the 296th to 317th amino acid) [9, 11]. Functionally, AT3 involves in protein homeostasis and degradation, myogenesis, cytoskeleton and transcription regulation, and cell cycle and death [7, 9–12]. In the past decades, the dysregulation of AT3 has been reported to play an important role in the pathogenesis of human chronic damage diseases such as a preponderance of evidence has implicated aberrant, gastric cancer, and breast cancer [10, 13–16]. Despite the collected data, the association between the genetic variables in the AT3 gene and AFB1-related chronic disease liver cirrhosis is still unclear. Here, we investigated whether genetic SNPs in this gene affect the risk of liver cirrhosis correlated with AFB1 exposure.

## RESULTS

### Baseline features of study subjects

(Table 1) summarized the baseline features (including demographic characteristics and AFB1 exposure information) of cases with liver cirrhosis and controls. Higher serum AFB1 albumin adducts (AAAs) were observed in the individuals with liver cirrhosis than these without liver cirrhosis (1.48 ± 0.32 vs. 2.40 ± 0.91 ln fmol/mg). Results from relative risk analyses based on multivariable regression model further showed that these individuals with medium AFB1 exposure featured a 3.59-time risk of liver cirrhosis compared to those with low exposure; whereas those with high exposure would face higher risk [adjusted odd ratio (OR), 7.74; 95% confidence intervals (CI), 5.51–10.87]. This indicates that the risk of liver cirrhosis was significantly positively correlated with the levels of AFB1 exposure. There was not significant difference for other baseline features.

**Table 1:** Demographic and etiologic characteristics of study subjects

Variable	Screening stage		Validation stage		Validation stage		
	Controls	LCs	Controls	LCs	Controls	LCs	OR (95% CI/*P*_trend_)^a^
Total	100	100	751	284	851	384	
Age (yrs)							
≤ 49	54	54	402	150	456	204	Reference
> 49	46	46	349	134	395	180	1.07 (0.83–1.40/0.59)
Gender							
Male	76	76	568	213	644	289	Reference
Female	24	24	183	71	207	95	1.32 (0.90–1.93/0.16)
Ethnicity							
Han	51	51	377	142	428	193	Reference
Zhuang	49	49	374	142	423	191	0.99 (0.77–1.29/0.98)
Smoking status							
No	52	52	390	132	442	184	Reference
Yes	48	48	361	152	409	200	1.34 (0.76–2.38/0.31)
Drinking status							
No	49	49	364	129	413	178	Reference
Yes	51	51	387	155	438	206	1.10 (0.81–1.44/0.75)
HBsAg							
Negative	62	62	462	168	524	230	Reference
Positive	38	38	289	116	327	154	1.08 (0.81–1.44/0.61)
Anti-HCV							
Negative	90	90	673	248	763	338	Reference
Positive	10	10	78	36	88	46	1.11 (0.71–1.74/0.64)
AFB1 exposure^b^							
Low	65	27	478	77	543	104	Reference
Medium	25	38	189	107	214	145	3.59 (2.65–4.84/8.54 × 10^-17^)
High	10	35	84	100	94	135	7.74 (5.51–10.87/4.46 × 10^-32^)

^a^OR conditional on matched set.

^b^The mean ± S.D. level of AFB1-album adducts is 1.48 ± 0.32 ln fmol/mg for controls and 2.40 ± 0.91 ln fmol/mg for cases with LC, respectively.

### AT3 rs8021276 polymorphism correlated with liver cirrhosis risk

According to the criteria of SNP selection, 264 common SNPs in the AT3 gene were tested in the screening groups. Analysis of the selected SNPs revealed the genotypic distributions in the control individuals were in Hardy–Weinberg equilibrium (data not shown). Among these selected SNPs, there SNPs (rs8021276, rs7158733, and rs10146249) were observed to significantly associate with liver cirrhosis risk in initial screening group (Table 2). Among individuals in the validation set, only rs8021276 significantly modified the risk of liver cirrhosis (OR = 3.09, 95% CI = 2.27–4.19, *P* = 5.33 × 10^-13^) according to the significance threshold (α_correct_ = 1.87 × 10^-4^). In the merged analyses of the screening and validation group (Table 2), the relative risk for liver cirrhosis among those with heterozygotes of rs8021276 A and G alleles (rs8021276-AG) versus those homozygotes of rs8021276 G alleles (rs8021276-GG) was 2.48 (1.84–3.33). The corresponding risk value for those homozygotes of rs8021276 G alleles (rs8021276-GG) was 6.98 (4.35–11.22). Thus, the risk of liver cirrhosis was positively related to the number of rs8021276 G alleles.

**Table 2:** Ataxin-3 polymorphisms and liver cirrhosis (LC) risk

	Screening set			Validation set			Merge set		
SNP	Controls (*n* = 100)	LCs (*n* = 100)	OR (95% CI/*P*_trend_)^a^	Controls (*n* = 751)	LCs (*n* = 284)	OR (95% CI/*P*_trend_)^a^	Controls (*n* = 851)	LCs (*n* = 384)	OR (95% CI/*P*_trend_)^a^
rs8021276									
AA	72	43	Reference	543	120	Reference	615	163	Reference
AG	24	35	2.97 (1.43–6.16/3.52 × 10^-3^)	178	100	2.45 (1.75–3.42/1.417 × 10^-7^)	202	135	2.48 (1.84–3.33/2.00 × 10^-9^)
GG	4	22	11.92 (3.42–41.55/9.97 × 10^-5^)	30	64	6.54 (3.85–11.10/3.49 × 10^-12^)	34	86	6.98 (4.35–11.22/9.27 × 10^-16^)
AG/GG	28	57	4.16 (2.11–8.18/3.62 × 10^-5^)	208	164	3.09 (2.27–4.19/5.33 × 10^-13^)	236	221	3.17 (2.42–4.16/8.08 × 10^-17^)
rs7158733									
GG	57	32	Reference	345	112	Reference	402	144	Reference
GT	30	35	3.59 (1.62–7.99/1.72 × 10^-3^)	242	97	1.25 (0.89–1.75/0.21)	272	132	1.41 (1.014–1.91/0.03)
TT	13	33	6.59 (2.57–16.88/8.57 × 10^-5^)	164	75	1.38 (0.95–2.00/0.10)	177	108	1.70 (1.22–2.37/1.78 × 10^-3^)
GT/TT	43	68	4.50 (2.18–9.31/4.98 × 10^-5^)	406	172	1.30 (0.96–1.75/0.09)	449	240	1.52 (1.17–1.99/2.03 × 10^-3^)
rs10146249									
CC	46	20	Reference	292	93	Reference	338	113	Reference
CA	41	48	3.08 (1.47–6.43/2.76 × 10^-3^)	288	141	1.72 (1.23–2.40/1.40 × 10^-3^)	329	189	1.96 (1.45–2.64/1.26 × 10^-4^)
AA	13	32	5.77 (2.31–14.37/1.59 × 10^-4^)	171	50	1.20 (0.78–1.83/0.41)	184	82	1.67 (1.16–2.41/5.80 × 10^-3^)
CA/AA	54	80	3.74 (1.87–7.49/1.86 × 10^-4^)	459	191	1.54 (1.13–2.09/6.24 × 10^-3^)	513	271	1.86 (1.41–2.46/1.58 × 10^-3^)

^a^OR conditional on matched set.

To explore whether the matching variables (including age, gender, ethnicity, drinking and smoking status, and HBV and HCV infection status) affects AT3 rs8021276 polymorphism modifying liver cirrhosis, we finished a series of stratified analyses according to these matching variables (Table 3). Similar risk significance was found among these different stratified variables, with an about 3.5-time risk value for rs8021276-AG/GG. Interactive analyses based on likelihood ratio tests further proved that matching variables did not affect AT3 rs8021276 polymorphism modifying liver cirrhosis risk (*P*_interact_ > 0.05).

**Table 3:** Ataxin-3 (AT3) rs8021276 polymorphism and liver cirrhosis (LC) risk stratified by matching factors

Variable	AT3	Controls (*n* = 851)		LCs (*n* = 384)		OR (95% CI)^i^	*P*_trend_
		*n*	%	*n*	%		
Age (yrs)^a^	rs8021276						
≤ 49	AA	330	72.4	88	43.1	Reference	
	AG	109	23.9	73	35.8	2.52 (1.72–3.68)	1.76 × 10^-6^
	GG	17	3.7	43	21.1	9.50 (5.17–17.47)	4.32 × 10^-13^
	AG/GG^h^	126	27.6	116	56.9	3.46 (2.45–4.89)	1.78 × 10^-12^
> 49	AA	285	72.2	75	41.7	Reference	
	AG	93	23.5	62	34.4	2.55 (1.69–3.84)	8.17 × 10^-6^
	GG	17	4.3	43	23.9	9.66 (5.21–17.92)	5.96 × 10^-13^
	AG/GG	110	27.8	105	58.3	3.65 (2.52–5.28)	7.09 × 10^-12^
Gender^b^	rs8021276						
Male	AA	465	72.2	123	42.6	Reference	
	AG	152	23.6	102	35.3	2.53 (1.84–3.49)	1.26 × 10^-6^
	GG	27	4.2	64	22.1	9.15 (5.57–15.02)	2.16 × 10^-18^
	AG/GG	179	27.8	166	57.4	3.51 (2.62–4.69)	3.67 × 10^-17^
Female	AA	150	72.5	40	42.1	Reference	
	AG	50	24.2	33	34.7	2.49 (1.42–4.38)	1.48 × 10^-3^
	GG	7	3.4	22	23.2	11.69 (4.66–29.35)	1.63 × 10^-7^
	AG/GG	57	27.5	55	57.9	3.64 (2.19–6.07)	6.68 × 10^-7^
Race^c^	rs8021276						
Han	AA	303	70.8	78	40.4	Reference	
	AG	102	23.8	65	33.7	2.47 (1.66–3.68)	8.51 × 10^-6^
	GG	23	5.4	50	25.9	8.47 (4.86–14.73)	4.30 × 10^-14^
	AG/GG	125	29.2	115	59.6	3.57 (2.51–5.10)	2.06 × 10^-12^
Zhuang	AA	312	73.8	85	44.5	Reference	
	AG	100	23.6	70	36.6	2.57 (1.74–3.79)	1.92 × 10^-6^
	GG	11	2.6	36	18.8	11.99 (5.85–24.60)	1.24 × 10^-11^
	AG/GG	111	26.2	106	55.5	3.51 (2.45–5.02)	6.83 × 10^-12^
Smoking^d^	rs8021276						
No	AA	332	75.1	95	46.6	Reference	
	AG	98	22.2	73	35.8	2.29 (1.22–4.68)	2.86 × 10^-9^
	GG	12	2.7	36	17.6	7.12 (3.51–16.44)	6.22 × 10^-13^
	AG/GG	110	24.9	109	53.4	3.37 (2.03–5.29)	2.47 × 10^-15^
Yes	AA	283	69.2	68	37.8	Reference	
	AG	104	25.4	62	34.4	1.92 (1.29–2.85)	1.24 × 10^-3^
	GG	22	5.4	50	27.8	7.31 (4.19–12.74)	2.26 × 10^-12^
	AG/GG	126	30.8	112	62.2	3.26 (2.42–4.05)	3.78 × 10^-9^
Drinking^e^	rs8021276						
No	AA	295	71.4	73	41.0	Reference	
	AG	99	24.0	60	33.7	2.45 (1.63–3.70)	1.90 × 10^-5^
	GG	19	4.6	45	25.3	9.75 (5.37–17.70)	7.32 × 10^-14^
	AG/GG	118	28.6	105	59.0	3.61 (2.50–5.21)	7.66 × 10^-12^
Yes	AA	320	73.1	90	43.7	Reference	
	AG	103	23.5	75	36.4	2.59 (1.77–3.78)	8.35 × 10^-7^
	GG	15	3.4	41	19.9	9.72 (5.15–18.36)	2.42 × 10^-12^
	AG/GG	118	26.9	116	56.3	3.47 (2.47–4.95)	1.55 × 10^-12^
HBsAg^f^	rs8021276						
Negative	AA	377	71.9	96	41.7	Reference	
	AG	125	23.9	84	36.5	2.62 (1.85–3.77)	9.08 × 10^-8^
	GG	22	4.2	50	21.7	8.92 (5.15–15.46)	5.71 × 10^-15^
	AG/GG	147	28.1	134	58.3	3.58 (2.59–4.95)	1.22 × 10^-14^
Positive	AA	238	72.8	67	43.5	Reference	
	AG	77	23.5	51	33.1	2.35 (1.51–3.68)	1.67 × 10^-4^
	GG	12	3.7	36	23.4	10.65 (5.25–21.60)	5.63 × 10^-11^
	AG/GG	89	27.2	87	56.5	3.47 (2.33–5.19)	1.17 × 10^-9^
Anti–HCV^g^	rs8021276						
Negative	AA	552	72.3	143	42.3	Reference	
	AG	181	23.7	117	34.6	2.50 (1.86–3.36)	1.50 × 10^-9^
	GG	30	3.9	78	23.1	10.03 (6.34–15.89)	7.92 × 10^-23^
	AG/GG	211	27.7	195	57.7	3.57 (2.73–4.66)	1.33 × 10^-20^
Positive	AA	63	71.6	20	43.5	Reference	
	AG	21	23.9	18	39.1	2.70 (1.21–6.04)	0.02
	GG	4	4.5	8	17.4	6.17 (1.67–22.80)	6.31 × 10^-3^
	AG/GG	25	28.4	26	56.5	3.25 (15.4–6.86)	1.92 × 10^-3^

^a^Likelihood ration test for interaction of the stratified variable (Age: ≤ 49 yrs and > 49 yrs) and AT3 genotype was calculated as test for the heterogeneity of ORs across strata (*P*_interaction_ = 0.722).

^b^Likelihood ration test for interaction of the stratified variable (female and male) and AT3 genotype was calculated as test for the heterogeneity of ORs across strata (*P*_interaction_ = 0.793).

^c^Likelihood ration test for interaction of the stratified variable (Han and Zhuang) and AT3 genotype was calculated as test for the heterogeneity of ORs across strata (*P*_interaction_ = 0.935).

^d^Likelihood ration test for interaction of the stratified variable (smoking status: no and yes) and AT3 genotype was calculated as test for the heterogeneity of ORs across strata (*P*_interaction_ = 0.643).

^e^Likelihood ration test for interaction of the stratified variable (drinking status: no and yes) and AT3 genotype was calculated as test for the heterogeneity of ORs across strata (*P*_interaction_ = 0.764).

^f^Likelihood ration test for interaction of the stratified variable (HBsAg–negative and positive) and AT3 genotype was calculated as test for the heterogeneity of ORs across strata (*P*_interaction_ = 0.894).

^g^Likelihood ration test for interaction of the stratified variable (anti-HCV-negative and positive) and AT3 genotype was calculated as test for the heterogeneity of ORs across strata (*P*_interaction_ = 0.793).

^h^AG/GG represented the genotypes with AT3 rs8021276 G allele.

^i^OR conditional on matched set.

### Interaction between AT3 rs8021276 polymorphism and AFB1 exposure

To explore the relationship between AFB1 exposure status and AT3 rs8021276 polymorphism in the risk for liver cirrhosis, we accomplished a joint analysis of AT3 rs8021276 genotypes and AFB1 exposure using individuals with both rs8021276-AA and low level of AFB1 exposure as a reference (OR = 1) (Table 4). The results indicated that higher levels of AFB1 exposure gradually increased liver cirrhosis risk (adjusted OR, from 1 to 6.30 among these with rs8021276-AA); the risk significance was more noticeable among subjects with the risk genotypes of AT3 rs8021276 (adjusted OR, from 1.88 to 11.46 for rs8021276-AG and from 4.27 to 34.01 for rs8021276-GG, respectively). This interaction of genotypes and AFB1 exposure was multiplicative according to gene-environment interaction formula [OR_eg_ > (OR_eg’_ × OR_e’g_)] [17].

**Table 4:** Joint effects of AFB1 exposure and rs8021276 polymorphism on liver cirrhosis (LC) risk

AFB1 exposure level/Genotype	Controls (*n* = 851)		LCs (*n* = 384)		OR (9% CI)^a^	*P*
	*n*	%	*n*	%		
Low/AA	398	46.8	57	14.8	Reference	
AG	134	15.7	36	9.4	1.88 (1.18–2.98)	7.43 × 10^-3^
GG	11	1.3	11	2.9	4.27 (1.92–9.50)	1.52 × 10^-5^
AG/GG^b^	145	17.0	47	12.2	2.27 (1.47–3.48)	1.98 × 10^-4^
Medium/AA	156	18.3	51	13.3	2.28 (1.50–3.48)	1.20 × 10^-4^
AG	43	5.1	58	15.1	9.42 (5.82–15.26)	7.93 × 10^-20^
GG	15	1.8	36	9.4	16.75 (8.63–32.52)	8.24 × 10^-17^
AG/GG	58	6.8	94	24.5	11.32 (7.37–17.38)	1.54 × 10^-28^
High/AA	61	7.2	55	14.3	6.30 (3.98–9.96)	3.51 × 10^-15^
AG	25	2.9	41	10.7	11.46 (6.48–20.26)	4.90 × 10^-17^
GG	8	0.9	39	10.2	34.01 (15.13–76.43)	1.41 × 10^-17^
AG/GG	33	3.9	80	20.8	16.93 (10.36–27.68)	1.58 × 10^-29^

^a^OR conditional on matched set.

^b^The combination of rs8021276 AG genotype and rs8021276 GG genotype.

### AT3 rs8021276 polymorphism affected AT3 expression and AFB1-DNA adduct levels

**Figure 1:**
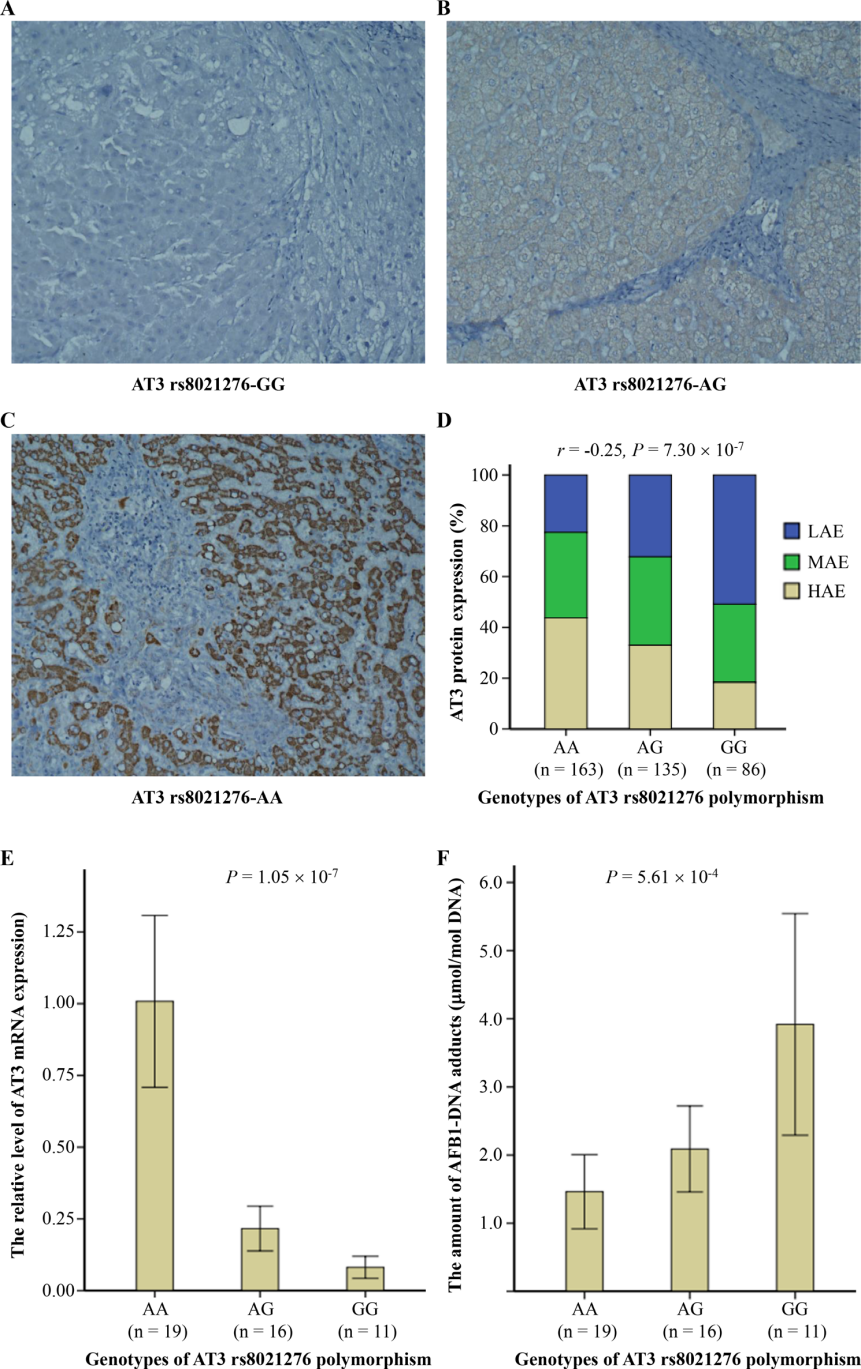
The ataxin-3 (AT3) rs8021276 polymorphism significantly correlated with AT3 expression and aflatoxin B1 (AFB1)-DNA adducts in the liver tissues with cirrhosis (**A**–**D**) The level of AT3 protein in liver tissue samples (*n* = 384) was tested by immunohistochemistry (IHC) and scored using the IHC scores of IRS system (*see Material and Methods*). Representative images show that different expression levels were observed in liver tissues from cases with different AT3 rs8021276 genotypes (scale bars: 50 mm) (**A**–**C**). The association between AT3 rs8021276 genotypes and AT3 protein levels was evaluated by spearman correlation test (**D**). E-F, Total RNAs and DNAs were extracted from fresh liver tissues with cirrhosis (*n* = 46), and use to test the levels of AT3 mRNA expression (**E**) and the amount of AFB1-DNA adducts (**F**) by TaqMan-PCR and comparative enzyme-linked immunosorbent assay. Data were analyzed using one-way ANOVA test (with Bonferroni correction). *Abbreviations:* LAE, low AT3 protein expression; MAE, medium AT3 protein expression; HAE, high AT3 protein expression.

To investigate whether the AT3 rs8021276 polymorphism affected the function of AT3, we conducted a relevant analysis between different genotypes of AT3 rs8021276 polymorphism and AT3 expression using immunohistochemistry (IHC) (Figure 1A–1D). We observed that these liver cirrhosis cases with different AT3 rs8021276 genotypes featured different expression levels of AT3 protein. Represent IHC images showed this expression difference (Figure 1A–1C). To analyze, the levels of AT3 protein expression was classed into low (IRS, < 4), medium (IRS, 4–8), and high expression (IRS, > 8), according to IHC scores of IRS systems [18]. Results from spearman's correlation analyses proved that the genotypes of AT3 rs8021276 was significantly negatively associated with the levels of AT3 protein (*r* = -0.25, *P* = 7.30´ 10^-7^) (Figure 1D). Similar results were observed in the analyses of AT3 mRNA expression and genotypes (Figure 1E). Next, we investigated the effects of AT3 rs8021276 polymorphism on the amount of AFB1-DNA adducts in the liver tissues with cirrhosis using comparative enzyme-linked immunosorbent assay and found that this polymorphism can modify the amount of DNA adducts (means ± S.D., 1.51 ± 1.23 μmol/mol DNA for rs8021276-AA, 2.09 ± 1.18 μmol/mol DNA for rs8021276-AG, and 3.92 ± 1.81 μmol/mol DNA for rs8021276-GG, respectively, *P* = 5.61 × 10^-4^) (Figure 1F). Taken together, these results were indicative of AT3 rs8021276 polymorphism modulating AT3 expression and the corresponding functions.

## DISCUSSION

In this study, we investigated the correlation between the genetic SNPs in the AT3 gene and liver cirrhosis risk among the Bose population who featured different degrees of AFB1 exposure. We found that AT3 rs8021276 polymorphism increased liver cirrhosis risk (OR = 2.48 for rs8021276-AG; 6.98 for rs8021276-GG). Furthermore, this polymorphism significantly interacted with environmental factor AFB1 exposure in the pathogenesis of liver cirrhosis. These results indicate that AT3 rs8021276 polymorphism may have functional significance in the pathogenesis of AFB1-related liver cirrhosis.

In China, more than ninety percent of liver cirrhosis results from chronic infection of hepatitis virus B (HBV) and C (HCV) [19–21]. A decrease in the frequency of hepatic virus infection as a major cause of this disease and an increase in the number of cases with liver cirrhosis caused by other causes (including AFB1) have recently been noted in China. AFB1 is an important chemical carcinogen that exerts its carcinogenicity through its metabolic activation to the reactive AFB1 8,9-exoepoxide by phase 1 cytochrome P450 monooxygenases [1, 22]. The reactive epoxide can bind to DNA and induce different degrees of DNA damage including adduction formation, and gene mutation [1, 2]. Our present study displayed that higher level of AFB1 was observed among these individuals with liver cirrhosis than among those without liver cirrhosis. This increasing exposure of AFB1 significantly affected the risk of liver cirrhosis, suggesting that AFB1 might play a crucial role in liver cirrhosis developing. In support of our findings, recent several studies have exhibited that the consumption of AFB1 can induce liver injury, inflammatory cell infiltration, fibrosis tissue proliferation, periportal fibrosis, and tissue cirrhosis [4, 5, 22–24]. The underlying mechanism of AFB1-induced liver cirrhosis remains unclear. However, some evidence from genetic susceptibility has recently been noted [25–28].

AT3 is well known for its role in the proteasomal protein degradation pathway, the maintenance of Ub recycling, and regulation of transcription via its deubiqutinating function. Previously, studies have suggested that the dysregulation of AT3 may involve in the pathogenesis of some human diseases including neurodegenerative disease and tumors [10, 13–16]. For example, more than 52 glutamines resulting from mutative expansion in the polyglutamine region of the C-terminus of AT3 protein will result in neurodegeneration and ultimately cause spinocerebellar ataxia 3 [7, 9, 10, 12]. Through a clinic-pathological study, Zeng *et al.*, found that AT3 protein and mRNA expression was lower in tumor tissues of gastric cancer than in peritumor tissues, and this down-regulating expression significantly affected clinic-pathological features of gastric cancer [14]. Sacco, *et al.* [13] and Ge, *et al.* [29], reported that the dysregulation of AT3 involved in the tumorigenesis of lung cancer and breast cancer. Our data also imply that genetic variants of AT3 promote the pathogenesis of liver cirrhosis. Taken together, these data are indicative of the vital role of AT3 gene in some human diseases.

Until now, over 2,000 genetic SNPs have been identified in the AT3 gene (*SNP database, PubMed*), some of which have reported to correlate with human diseases [30]. In the first stage of this study, a total of 264 known SNPs were selected for elucidating potential effects of genetic SNPs in the AT3 on liver cirrhosis risk. This was done mainly because the genetic variants may affect the structure and expression of AT3, and potentially involve in liver cirrhosis pathogenesis. Of these selected SNPs, only one (rs8021276) was identified to increased liver cirrhosis risk through two stage analyses (namely the screening stage and the validation stage). Furthermore, the rs8021276 was also observed to multiplicatively interact with the levels of AFB1 exposure in the pathogenesis of liver cirrhosis. Together, these data indicate that AT3 rs8021276 polymorphism may be a vital genetic susceptibility factor for liver cirrhosis. Although we observed some evidence of other SNPs, including rs7158733 and rs10146249, increasing liver cirrhosis risk, they were excluded in the final analyses according to the significance threshold (α_correct_ = 1.87 × 10^-4^) in the present study. Thus, the potential role of genetic SNPs in the AT3 gene influencing liver cirrhosis risk should not be neglected.

To explore the effect of AT3 rs8021276 polymorphism on AFB1-related liver cirrhosis, AFB1-DNA adducts and expression of AT3 of tissues samples with liver cirrhosis were examined. It was found that this polymorphism was correlated with increasing amount of DNA adducts and the downregulation of AT3 expression. A recent study has shown that this gene can modify DNA repair response to double strand damage of DNA [31]. The interactive analysis further indicated that AT3 rs8021276 polymorphism multiplicatively interacted with environmental variable AFB1 exposure, highlighting the possible role of this polymorphism in the predictive value for AFB1-induced liver cirrhosis. Collectively, these data imply that AT3 rs8021276 polymorphism may be linked to the lost or reduction of DNA repair ability via regulating AT3 expression. Therefore, it may display a vital role in the pathogenesis of liver cirrhosis related to AFB1 exposure.

For investigating the correlation between genetic SNPs and AFB1-related liver damage, it is very important to select sufficiently significant SNPs and elucidate AFB1 exposure levels [32–34]. In this study, we used a two-stage and individually matched design for screening and validating significant SNP sites and avoiding the effects of known confounders (such as the infective status of HBV and HCV). Results from multivariable and stratified analyses indicated that positive SNPs were significantly screened. Furthermore, confounders were effectively controlled and did not modify the relationship between the AT3 rs8021276 polymorphism and liver cirrhosis related to AFB1 exposure. Given that serum AAA marker is characterized by high stability and can reflect long-term exposure information of AFB1 [32], this marker was used to evaluate the AFB1 exposure status. Results also showed higher serum level of AAA was found in these cases with liver cirrhosis and increasing AAA level significant increased the risk of liver cirrhosis.

To conclude, our study firstly described the association between genetic polymorphisms in the AT3 gene and liver cirrhosis caused by AFB1 exposure, and our results provide some important evidence of AT3 rs8021276 polymorphism as a biologic marker for estimating the risk of liver cirrhosis caused by AFB1 exposure. In view of the fact that AT3 is one of major DUBs in response to cellular physiology by regulating both cytosolic and nuclear processes [10, 31], the present study provides new data for identifying high-risk individuals, especially these with high risk factor AFB1 exposure. However, the main important limitations of our study were potential selection bias (resulting from the hospital-based design) and possible biased distribution of liver disease severity (e.g., the HBV DNA level). Additionally, the function analyses of AT3 rs8021276 polymorphism affecting the risk of AFB1-related liver cirrhosis, except for the effects of this polymorphism on AT3 expression and AFB1-DNA adducts, were not finished. Therefore, detailed functional analyses combining AFB1 on the basis of a large samples may improve the present study.

## MATERIALS AND METHODS

### Study population

We utilized a hospital-based case–control study of AFB1-related liver cirrhosis in Bose, a knowledge high AFB1 exposure area from China. Liver cirrhosis patients who underwent liver biopsy in hospitals affiliated with Youjiang Medical University for Nationalities in Bose from January 2008 to December 2012 were utilized. Control individuals without clinic evidence of hepatic diseases were recruited from the general-health check-up center of the same hospitals, individual-matched (1:1 or 2:1) with cases by sex, age (± 5 years), ethnicity, and HBV and HCV infection. All controls were surveyed to ascertain their willingness to participate in the study and to provide preliminary demographic data. In this study, a total of 384 cases with liver cirrhosis and 851 controls, representing 93% of eligible cases and 95% of eligible controls were interviewed and included the final analysis. Informed consent was obtained from each participant before inclusion in the study. Subject evaluation included a structured interview that elicited information on demographic characteristics (including age, race, smoking and drinking status, and detailed medical history for themselves and their families); collection of study samples; and a standardized clinical examination. At the same time, 5 ml of peripheral blood was obtained for the analysis of AFB1 exposure and SNP genotypes along with demographic data. Forty-six fresh liver tissue specimens with liver cirrhosis were also collected for AT3 mRNA expression and AFB1-DNA adduct analysis. Among these subjects, we randomly selected 100-matched cases and controls to form the screening set and the remaining patients formed the validation set. This study was approved by the Institutional Ethics Committee of Youjiang Medical University for Nationalities, and was carried out in accordance with the approved guidelines (No. AYJM20071220).

### AFB1 exposure data

In this study, AFB1 exposure information was valuated using serum AAA levels of peripheral blood as previously published [35]. For statistical analysis, values were logarithmically transformed and then were divided into three subgroups: low (< 1.48 ln fmol/mg), medium (1.48–2.40 ln fmol/mg), and high (> 2.40 ln fmol/mg), according to the mean logit value of serum AAA among controls and cases.

### AFB1-DNA adducts assay

The DNA was extracted from fresh liver tissues with liver cirrhosis for AFB1-DNA adduct analysis. AFB1-DNA adducts levels was tested using comparative enzyme-linked immunosorbent assay as our previously described [36, 37].

### SNP selection and genotyping

In the screening study, we included 264 common (minor allele frequency > 0.1 in Asians) SNPs located in AT3 gene. Genotyping was performed by using SNaPshot method (Applied Biosystems [ABI], Foster City, CA), according to the manufacturers’ instructions. For genotypic analyses of SNPs, the controls and cases were genotyped at the same time, and duplicate test samples and two water samples (PCR negative controls) were included in each 96-well plate, with the technician blinded to the identity of the samples. The rate of concordant results in 100 duplicate samples was > 99%. In the validation-stage study, three significant SNPs (rs8021276, rs7158733, and rs10146249) were genotyped by the TaqMan polymerase chain reaction (TaqMan-PCR) assays using the ABI 7900 System. Each PCR was carried out in a total volume of 5 μL consisting of 1 ´ Premix Ex Taq™ (TaKaRa Biotechnology (Dalian) Co., Ltd., Dalian, China), 0.2 μM of each probe and each primer (analyzing# C_44789227_10, C_27204300_20, and C_30018528_10 for rs8021276, rs7158733, and rs10146249; cat# 4351379, ABI), and 10–20 ng of genomic DNA. The PCR program had an initial denaturation step of 10 min at 95°C followed by 40 cycles of 15 sec at 95°C and 1 min at 60°C. For quality control, controls were included in each run, and repeated genotyping and sequencing of a random 5% subset yielded 100% identical genotypes.

### Expression assays of AT3 protein

The levels of AT3 protein in formalin-fixed biopsy tissue samples with liver cirrhosis (*n* = 384) was tested by IHC technique as our previously described [35, 36, 38], and scored using the IHC scores of IRS system [18]. To analyze, the IRS amount of AT3 protein was grouped into three levels: low (IRS value, < 4), medium (IRS value, 4–8), and high expression (IRS value, > 8).

### Expression assays of AT3 mRNA

Total RNA was extracted from fresh tissues with liver cirrhosis using EZMA™ MicroElute Total RNA Kit with DNase I (Omega, Norcross, GA) and corresponding first-strand cDNA was synthesized using RevertAid™ First Strand cDNA Synthesis Kit (Fermentas, Glen Burnie, MD). The relative quantitation of AT3 mRNA-expressing levels using the comparative CT method (2^-ΔΔCt^ method) was carried out by TaqMan-PCR (with an internal control UBC mRNA). The primers were: 5’-GAGGA TGAGA ATGGC AGAAG GA-3’ and 5’-CCAGA AGGCT GCTGT AAAAA CG-3’for AT3 and 5’-GGGCA CTGGT TTTCT TTCCA-3’ and 5’-CGCCG AGAAG GGACT ACTTT T-3’ for UBC (Shanghai GeneCore BioTechnologies Co., Ltd., Shanghai, China) [35]. The probes were: 5’-FAM-AGTTA CTAGT GAAGA TTATC G-MGB-3’ for AT3 and 5’- HEX-AGAGCGGAACAGGC-MGB-3’ for UBC (Shanghai GeneCore BioTechnologies Co., Ltd.) [35]. PCR amplification was performed in a 25-μL final reaction mixture containing 1 × Premix Ex Taq™ (TaKaRa), 0.2 μM of each probe, 0.2 μM of each primer, and 1 μL reverse transcription product with cDNAs. PCR reaction conditions comprised an initial step at 95°C for 2 min, followed by 45 cycles at 95°C for 10 sec and 60°C for 1 min. Data analysis for the relative level of AT3 mRNA expression was performed with the Bio-Rad iQ™ Optical System Software Version 2.0.

### Statistical analysis

The χ2 test, Student *t* test, or one-way ANOVA test was used to test for differences between groups, demographic characteristics, AFB1-exposure information, and SNP genotypes. Conditional logistic regression was used to calculate ORs for risk of liver cirrhosis and their 95% CIs. In this study, the additive model (treating genotype as an ordinal variable) was used to screen the main effects of 264 SNPs in the AT3 for liver cirrhosis risk. The correlation matrix-based method was used to correct for multiple testing and 2-sided *P* values smaller than 1.87 × 10^-4^ were considered significant for the main effects of SNPs in the screening stage. In the joint analysis stage of SNPs and AFB1 exposure, genotype frequencies in these groups were further adjusted for multiple comparisons using Bonferroni's method, and the significance threshold was lowered to α_correct_ = 9.35 × 10^-5^. All statistical analyses were done using the statistical package for social science (SPSS) version 18 (SPSS Institute, Chicago, IL).

## Abbreviations

AT3: ataxin-3
rs8021276-AA: the homozygote of rs8021276 A alleles
rs8021276-GG: the homozygote of rs8021276 G alleles
rs8021276-AG: the heterozygote of rs8021276 G alleles
rs8021276-AG/GG: genotypes with rs8021276 A and G alleles
